# Astragaloside IV Downregulates *β*-Catenin in Rat Keratinocytes to Counter LiCl-Induced Inhibition of Proliferation and Migration

**DOI:** 10.1155/2012/956107

**Published:** 2012-05-28

**Authors:** Fu-Lun Li, Xin Li, Yi-Fei Wang, Xiu-Li Xiao, Rong Xu, Jie Chen, Bin Fan, Wen-bin Xu, Lin Geng, Bin Li

**Affiliations:** ^1^Department of Dermatology, Yueyang Hospital of Integrated Traditional Chinese and Western Medicine, Shanghai University of Traditional Chinese Medicine, Shanghai 200437, China; ^2^Baoshan Branch, Shuguang Hospital, Shanghai University of Traditional Chinese Medicine and Pharmacy, Shanghai 201900, China

## Abstract

Re-epithelialization is a crucial step towards wound healing. The traditional Chinese medicine, *Astragalus membranaceus* (Fisch) Bge, has been used for hundreds of years for many kinds of ulcerated wounds. Recent research has identified the active compound in this drug as astragaloside IV (AS-IV), but the underlying molecular mechanisms of its therapeutic action on keratinocytes remain poorly understood. In this study, we used an *in vitro *model of ulcer-like wound processes, lithium chloride (LiCl)-induced cultured mouse keratinocytes, to investigate the effects of AS-IV treatment. The effects on cell proliferation were evaluated by the MTS/PMS colorimetric assay, effects on cell migration were determined by a wound-healing scratch experiment, effects on the cell cycle were analyzed by flow cytometry, and effects on protein expression were analyzed by immunoblotting and immunofluorescence. LiCl strongly inhibited cell proliferation and migration, up-regulated **β**-catenin expression, and down-regulated proliferating cell nuclear antigen (PCNA) expression. AS-IV treatment attenuat the inhibition of proliferation and migration, significantly reducing the enhanced **β**-catenin expression, and recovering PCNA and **β**-tubulin expression. Thus, AS-IV mediates mouse keratinocyte proliferation and migration *via *regulation of the Wnt signaling pathway. Down-regulating **β**-catenin to increase keratinocyte migration and proliferation is one mechanism by which AS-IV can promote ulcerated wound healing.

## 1. Introduction

The skin of all animals provides a protective barrier against entry by pathogens and environmental toxins. Skin cells also encompass a rich molecular infrastructure that involves immune system processes, which further defend against disease or injury to the underlying organs. Thus, cutaneous wounds not only affect skin homeostasis, but also represent a systemic threat; thus, the wound healing process is critical for general health and well-being [1]. The wound healing process is intricate and not yet fully understood; however, it is known that many underlying conditions, such as diabetes, cancer, and vascular disease, can prolong or block the process. In those cases, normal trauma caused by a simple accident or fall can become an ulcerated chronic wound, significantly impacting one's quality of life and increasing the risk of death.

 For many centuries, the world's physicians have sought out methods and therapeutic agents that will promote and accelerate wound healing [2]. Generally, the cutaneous wound healing process can be divided into three overlapping, but distinct, phases: the inflammatory phase, the proliferative phase (neoangiogenesis, tissue formation, reepithelialization) and the tissue remodeling phase [3–5]. Reepithelialization is considered a crucial step towards wound healing, and impairment of this process has been demonstrated to result in the development of chronic wounds [6].

**Figure 1:**
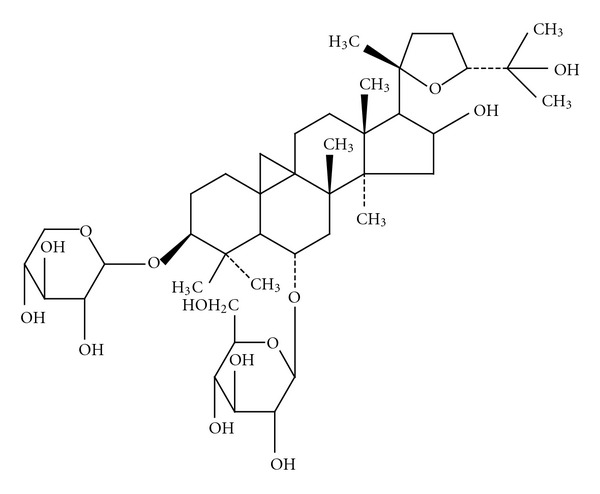
Chemical structure of astragaloside IV.

 The traditional Chinese medicine, *Astragalus membranaceus* (Fisch) Bge, has long been used to treat ulcerated wounds. Recently, the main active compound from this drug, astragaloside IV (AS-IV), a 3-O-*β*-D-xylopyranosyl-6-O-*β*-D-glucopyranosylcycloastragenol (chemical structure shown in Figure 1), was purified. Research studies into the biological properties of AS-IV have revealed strong anti-inflammatory activity, which involves inhibition of NF-*κ*B activation and downregulation of adhesion molecule expression [7]. In addition, many other pharmacological activities have been demonstrated, including antidiabetic and antioxidative activities [8]. However, the precise mechanisms by which AS-IV promotes wound healing remain to be elucidated. In order to develop this Chinese herb as a *bona fide* therapeutic agent to treat aberrant wound healing, it is necessary to gain a detailed understanding of the involved cellular and molecular mechanisms.

 It is known that activation of *β*-catenin/c-myc pathways can inhibit keratinocyte migration and differentiation, thereby impairing wound healing [9]. Prior studies using embryological models indicated that administration of lithium chloride (LiCl) can induce signaling pathways that mimic Wnt/*β*-catenin activation. Specifically, LiCl treatment inhibits GSK-3*β* activity by inducing phosphorylation at Ser-9 [10], which in turn, stabilizes free cytosolic *β*-catenin. Thus, *in vitro* treatment of cultured keratinocytes with LiCl allows for the controlled experimental analysis of the molecular processes underlying impeded wound healing.

 Activated *β*-catenin and c-myc have been clinically detected in the epidermis of chronic wounds. As such, it has been proposed that these molecules may serve as markers of impaired healing and may represent useful targets of therapeutic intervention [9]. In a previous study, we demonstrated that diabetic skin ulcers can be efficiently managed by treatment with a Chinese medicine cocktail that includes *Astragalus membranaceus* (Fisch) Bge. We hypothesized that the mechanism of action might involve inhibition of the Wnt signaling pathway [11]. The study presented herein was designed to investigate the potential effects of AS-IV on Wnt/*β*-catenin signaling in primary keratinocytes under LiCl-induced conditions. These findings reveal a positive role for AS-IV in cutaneous wound healing via downregulation of *β*-catenin expression.

## 2. Materials and Methods

### 2.1. Chemicals and Reagents

AS-IV (99.2% purity) was purchased from the National Institute for the Control of Pharmaceutical and Biological Products (Shanghai, People's Republic of China) and completely dissolved in room temperature DMSO at a stock concentration of 20 mg/mL. DAPI was purchased from Roche Diagnostic Corp. (Indianapolis, IN, USA). Bromodeoxyuridine (BrdU) detection kit was purchased from BD Biosciences (San Diego, CA, USA). LiC1 (pro analysis grade) was from Sigma-Aldrich (St. Louis, MO, USA). Antibodies against *β*-catenin and GAPDH were purchased from Cell Signaling Technology (Beverly, MA, USA). Guinea pig anti-K14 was purchased from RDI-Fitzgerald (Oaklyn, NJ, USA). Antibody against *β*-tubulin was purchased from Sigma-Aldrich. Immunofluorescence secondary antibodies, Alexa Fluor 488-conjugated (green) and Alexa Fluor 594-conjugated (red), were from Invitrogen (Carlsbad, CA, USA). Fetal calf serum (FCS) and other cell culture reagents, unless otherwise indicated, were from Gibco (Invitrogen).

### 2.2. Cell Culture

Primary mouse keratinocytes were prepared from newborn mice, as previously described [12], and cultured at 37°C in an atmosphere of 5% CO_2_ in Cnt-07 medium with appropriate supplements (Cellntec, Berne, Switzerland).

### 2.3. Cell Proliferation (MTS/PMS) and BrdU Incorporation Assays

Cell proliferation was assayed using the MTS/PMS method. Briefly, cells (1 × 10^3^/well) were plated in 96-well culture plates (Nunclon; Nunc, Roskide, Denmark). After overnight incubation, cells were treated in the absence or presence of LiCl and AS-IV. Each concentration was regarded as a single treatment group, while the control group only contained DMSO. Each group contained six replicate wells. After culture plates were incubated for 0, 24, 48, or 72 h, 100 *μ*L of MTS/PMS working solution was added to each well, and the keratinocytes were then incubated continuously for another 4 h. The absorbance (*A* value) of each well was measured using a spectrophotometer (Beckman Coulter; Miami, FL, USA) at 490 nm. The effect of AS-IV on the cell growth inhibitory rate in keratinocytes was calculated according to the following formula: % inhibitory rate = [A_490_ value of treated group]/[( A_490_ value of control group) × (100%)]. The 50% inhibitory concentration (IC_50_) was determined from dose-response data from at least three independent experiments. Cell number and cell viability were determined daily using a Coulter Counter (Beckman Coulter-Z2) and the trypan blue dye exclusion test. For the 5-bromo-2′-deoxyuridine (BrdU) incorporation assay, cells were seeded and serum starved as above. After treatment with PBS or LiCl at different concentrations for 12 h in 10% FBS-containing media, cells were incubated with BrdU for an additional 2 h. BrdU-labeled cells were detected using an enzyme-linked immunosorbent assay- (ELISA-) based colormeric kit (BD Biosciences; San Diego, CA, USA) according to the protocol provided by the manufacturer. 

### 2.4. Western Blot Analysis

 Lysates from cultured cells were prepared as previously described [13]. Whole cell protein extracts were resolved by SDS-PAGE and electrotransferred to membranes for specific antibody detection as previously described [14].

### 2.5. *In Vitro* Scratch Assay

When mouse keratinocytes reached 100% confluency, cells were transferred to basal KBM medium (Life Technologies, Inc., Grand Island, NY, USA) and incubated for 24 h. Cells were then treated with 8 *μ*g/mL mitomycin (ICN, Irvine, CA, USA) for 1 h and then washed with basal media. LiCl, an inhibitor of glycogen synthase kinase 3 (GSK3), was applied to activate Wnt signaling. Since LiCl can also efficiently block EGF-stimulated migration, EGF-induced cell migration was used as a positive control. Scratches were performed in treated cell cultures as previously described, and cultures were photographed. Then, mixtures of 20 *μ*mol/L LiCl with or without 25 ng/mL EGF and 80 *μ*g/mL AS-IV were added to the cultures and incubated for 24 h, 48 h, or 72 h. The cells were rephotographed, and comparisons to the pre-treatment images were used to quantitatively determine the extent of cell migration as previously described. Briefly, the distance covered by a cell moving into the scratch wound area was measured. Thirty measurements were taken for each experimental condition. Three images were analyzed per condition and per time point. 

### 2.6. Flow Cytometry for Cell Cycle Analysis

Cells were plated at a density of 1.5 × 10^6^ cells per 6 cm diameter dish and allowed to grow for 24 h, after which the medium was changed to serum-free medium. After 16–18 h of starvation, cells are synchronized in the G_0_ phase of the cell cycle. Different experimental reagents were then added to the serum-free medium and incubated for the indicated times. Cells (1 × 10^6^) were harvested by trypsinization, washed twice in cold PBS, and fixed in 70% alcohol for 30 min on ice. After washing in cold PBS three times, cells were resuspended in 1 mL Krishan staining solution [15] and left overnight at 4°C. The next day, cells were filtered through a 96-micron pore size nylon mesh, and a total of 10000 stained nuclei were analyzed with a flow cytometer (BD Biosciences). DNA histograms were prepared using the accompanying ModFit analysis program (BD Biosciences), which plots and statistically fits the fractions of cells in the G_0_-G_1_, S, and G_2_-M phases. Each condition was repeated in triplicate.

### 2.7. Immunofluorescence Staining

Cells were cultured on 8-well chamber slides (Thermo Fisher Scientific, Waltham, MA, USA) and treated with different concentrations of AS-IV for 24 h. Cells were then fixed with 4% paraformaldehyde, permeabilized with 0.3% Triton X-100, and digested in pepsin solution (Lab Vision, Thermo Fisher Scientific). Nonspecific binding in crude protein extracts was blocked by incubating cells in 10% normal goat serum plus 0.1% NP-40 for 1 h. Detection of specific proteins was carried out by incubating with primary antibodies overnight. Alexa Fluor 488- or 594-conjugated secondary antibodies were used for immunofluorescence staining, and images were obtained with a Leica SP2 confocal microscope (Deerfield, IL, USA).

### 2.8. Statistical Analysis

Data are presented as the mean ± SD of at least three independent experiments, unless designated otherwise. Statistical analysis was carried out using Student's *t-*Test, and a value of *P* < 0.05 was considered to be significant.

## 3. Results

### 3.1. AS-IV Attenuates LiCl-Induced Inhibition of Keratinoctye Growth

**Figure 2:**
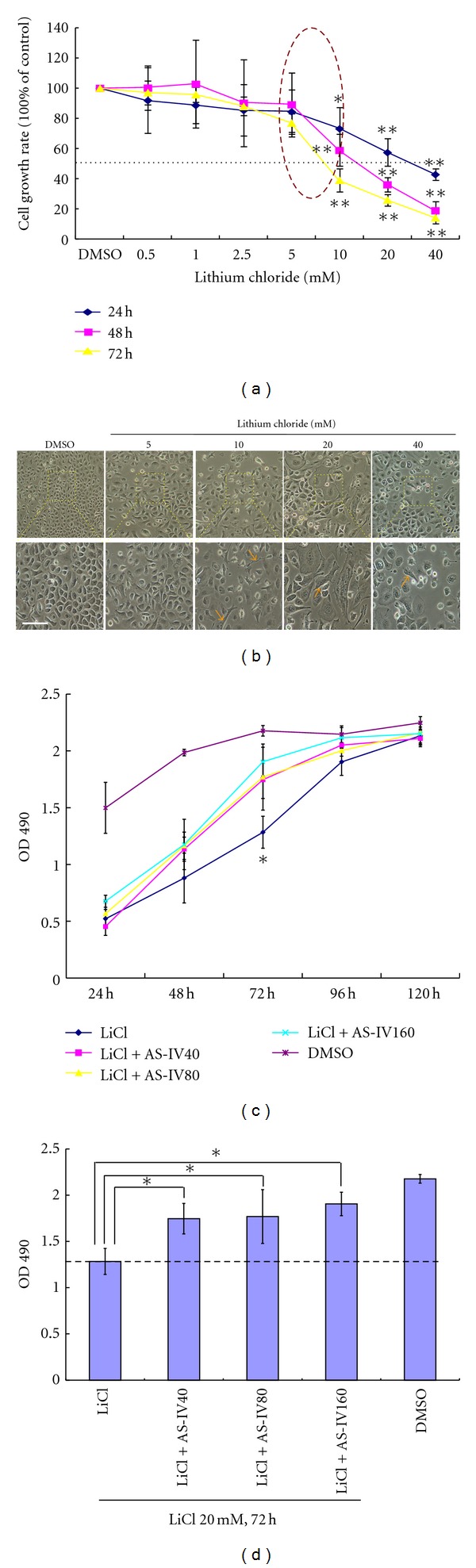
Cell proliferation of keratinocytes in response to LiCl and AS-IV treatment. (a) LiCl inhibits proliferation of mouse keratinocytes, as evidenced by the MTS/PMS assay. Data are expressed as a percentage of the control (100%) and represent the mean ± SD of three experiments. *P < 0.05, **P < 0.01. (b) LiCl induced a senescent-like morphology in live keratinocytes, as evidenced by phase-contrast microscopy. (c) Growth curve of keratinocytes in response to LiCl and AS-IV treatments. (d) OD at 490 nm of keratinocytes treated with or without LiCl and AS-IV for 72 h. Scale bar = 100 *μ*m.

**Figure 3:**
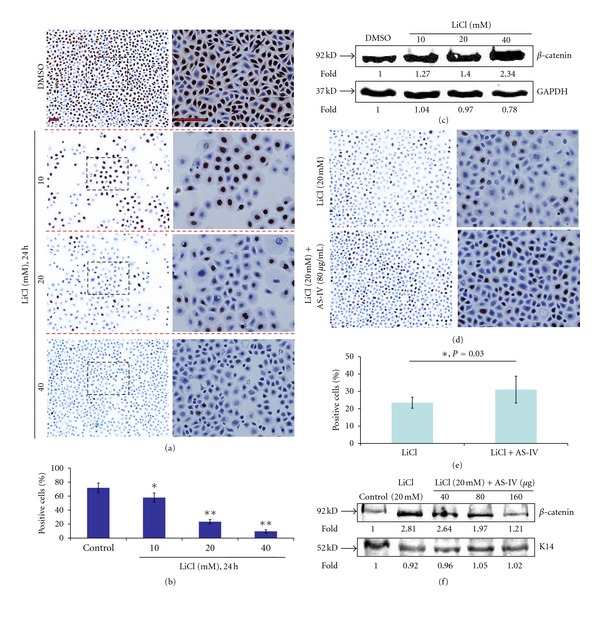
BrdU staining of cells treated with LiCl in the absence of presence of AS-IV. (a) LiCl treatment produced a concentration-dependent decrease in BrdU-positive cells compared to control cells. (b) Histograms represent the mean ± SD of the percent of BrdU-positive cells treated with different concentrations of LiCl. (c) Changes in *β*-catenin protein expression in response to LiCl. (d, e, f) Comparison of the effects of LiCl ± AS-IV on cell proliferation, by detection of BrdU incorporation and *β*-catenin protein expression. AS-IV resolved LiCl-induced inhibition of cell proliferation and downregulation of *β*-catenin. *P < 0.05. Expression of K14 and GAPDH were detected as protein loading controls Scale bar = 100 *μ*m.

**Figure 4:**
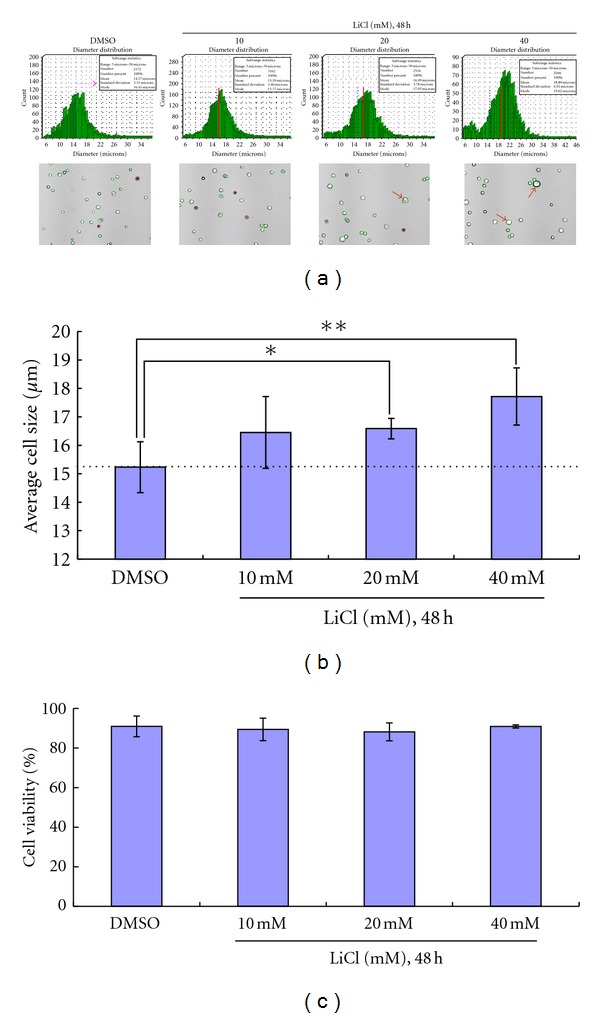
LiCl affects keratinocyte morphology but not cell viability. Treatment with 20 mM and 40 mM LiCl caused cell size to enlarge (a, red arrow). (b) Average cell size from three experiments. (c) LiCl treatment had no effect on keratinocyte viability, as evidenced by the cell viability assay.

To investigate the normal function of the Wnt signaling pathway in keratinocytes, we used LiCl to activate the Wnt/*β*-catenin signaling pathway. The effect of LiCl on keratinocyte proliferation was examined by the MTS/PMS assay and measurement of BrdU incorporation into DNA during DNA synthesis. As shown in Figure 2(a), keratinocytes were treated with different concentrations (0~40.0 mM/L) of LiCl for 24, 48, and 72 h. Controls were subconfluent and synchronous keratinocytes were treated with DMSO solvent alone for the indicated times. Compared to the control group, LiCl-treated keratinocytes exhibited consistent growth retardation. The half maximal (50%) inhibitory concentration (IC_50_) values (Figure 2(a), dotted line) of growth achieved after LiCl-treatment for 24, 48, and 72 h were 25.85 ± 5.03, 16.16 ± 0.95, and 12.35 ± 1.38 mM, respectively. Lower concentrations of LiCl (<5 mM) did not inhibit keratinocyte growth. Higher concentrations of LiCl (>5 mM) produced significant and dose-dependent inhibition of keratinocyte proliferation (Figure 2(a), circle). The BrdU incorporation assay also revealed a concentration-dependent decrease in BrdU-positive cells in response to different dosages of LiCl. As shown in Figure 3(a), the amount of BrdU-positive cells following LiCl-treatment of 10 mM (57.91 ± 6.57%), 20 mM (23.48 ± 3.19%), and 40 mM (9.67 ± 2.45%) was significantly less than those in the DMSO-treatment control group (71.81 ± 6.79%; all *P* < 0.01). Cell viability was unaffected by LiCl-treatment at all doses (Figure 4(c)). These data unambiguously confirm that LiCl is able to exert potent growth inhibitory effects on keratinocytes in a concentration- and time-dependent manner. These results also indicate that LiCl-treatment inhibits keratinocyte proliferation without affecting their viability. 

 To investigate the function of AS-IV in keratinocytes overexpressing *β*-catenin, we incubated the cells with AS-IV and LiCl for 72 h. As shown in Figures 2(c) and 2(d), AS-IV treatment attenuated LiCl-induced inhibition of cell proliferation, as evidenced by the fact that optical density (OD) at 490 nm was significantly higher in the LiCl group than in the DMSO control group (*P* < 0.05). Similarly, the BrdU assay showed that AS-IV treatment was able to enhance the percentage of positive cells (from 23.48% to 31.05%; Figure 3(e)), suggesting that AS-IV was able to promote DNA synthesis. 

### 3.2. LiCl Induces Senescence-Like Changes in Keratinocytes

LiCl treatment also led to obvious morphological changes in keratinocytes, including increased size, spreading, and flattened appearance. There were no rounded cells, characteristic of apoptosis, observed in LiCl-treated cultures (Figure 2(b)). Using the cell viability assay, we measured the change in diameter of keratinocytes in response to exposure to different doses of LiCl (from 10 mM to 40 mM) for 48 h. As shown in Figure 4, LiCl treatment at doses of 20 mM and 40 mM resulted in an enlarged cell size (Figure 4(a), red arrow), which indicated that high concentrations of LiCl may change keratinocyte morphology. The average diameters of LiCl-treated keratinocytes were greater (10 mM, 16.45 ± 1.36 *μ*m; 20 mM, 16.59 ± 0.36 *μ*m; 40 mM, 17.72 ± 1.01 *μ*m) than that in the control group (15.23 ± 0.97 *μ*m).

### 3.3. AS-IV Attenuates LiCl-Induced S Phase Cell Cycle Arrest in Keratinocytes

**Figure 5:**
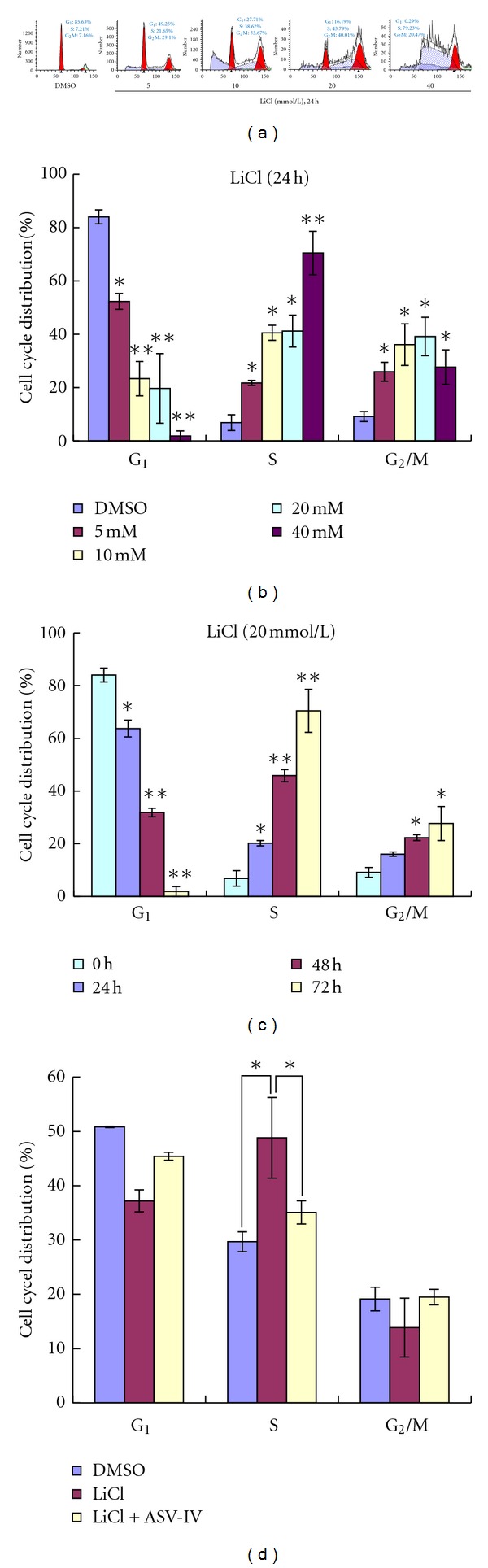
LiCl induces S phase arrest in keratinocytes. (a) Keratinocytes were exposed to various concentrations of LiCl for 24 h. (b) Keratinocytes were exposed to 20 mM LiCl for 24 h, 48 h, and 72 h. (c) Histogram showing the average amounts of cells in various cell cycle stages from three experiments following a 24 h exposure to different LiCl concentrations. (d) Histogram showing the average distribution of cells in various cell cycle stages from three experiments with LiCl ± AS-IV, and LiCl + EGF treatments.

LiCl-induced inhibition of growth may result from cell cycle effects. Thus, the cell cycle distribution of LiCl-treated and control cells was determined by measuring the DNA content of Krishan-stained cells using fluorescence-activated cell sorting. As shown in Figure 5, LiCl-treated cells exhibited a higher proportion of cells in the S phase than control cells. Histograms were generated to visualize the percent of cell cycle distributions produced by different LiCl concentrations (Figure 5(b)) and different exposure times (Figure 5(c)). Interestingly, when LiCl-treated cells were exposed to AS-IV, there was remarkable attenuation of induced cell cycle arrest in the S phase. Specifically, the percentage of S phase cells in LiCl-treated keratinocytes cultures went from 48.81 ± 7.43 to 35.28 ± 2.14 upon AS-IV exposure (*P* < 0.05). 

### 3.4. AS-IV Attenuates LiCl-Induced Overexpression of *β*-Catenin in Keratinocytes

**Figure 6:**
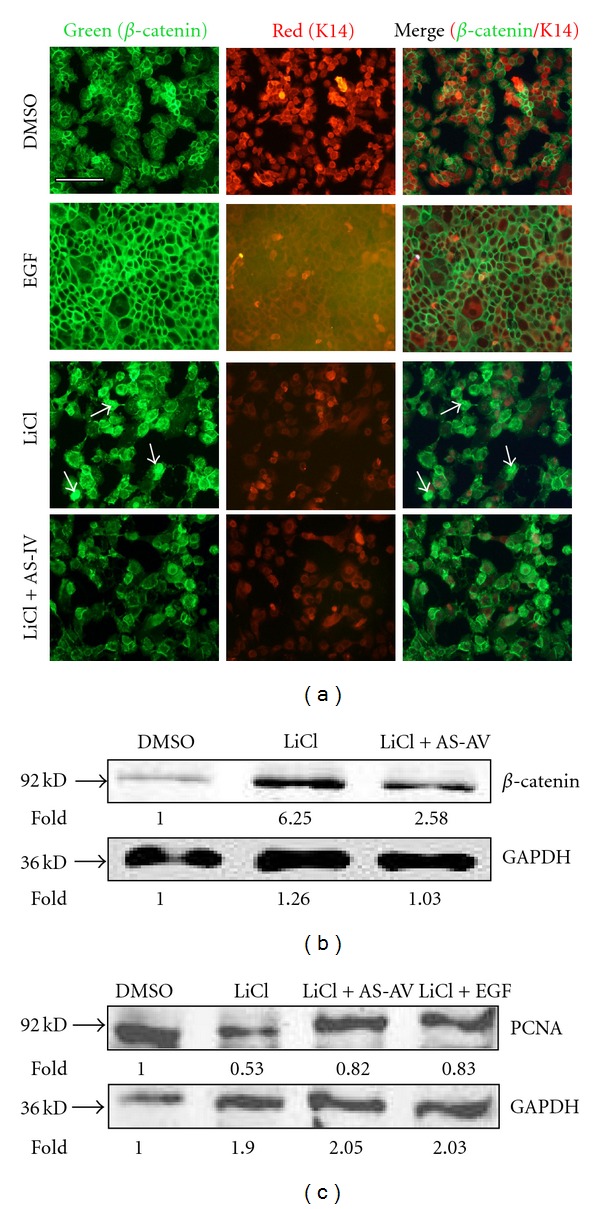
*β*-catenin is differentially regulated by LiCl and AS-IV. Stabilization of *β*-catenin in keratinocytes was followed by immunofluorescence staining with a *β*-catenin-specific antibody (a) and Western blot analysis (b). (a) DMSO (negative control) and EGF (positive control) treated keratinocytes exhibited normal *β*-catenin membrane expression. The LiCl-treated group showed stabilized *β*-catenin, which was downregulated following AS-IV treatment. (b) GAPDH protein expression was used as the protein loading control. (c) Western blot analysis revealed that PCNA expression, which is related to cell proliferation, was downregulated 50% by LiCl compared to the control cells, and upregulated to 80% by AS-IV. GAPDH expression was used as a protein loading control. Asterisks indicate statistically significant differences between control and LiCl-treated cells. The scale bar in the first panel of (a) represents 100 *μ*m, and is applicable to both sections.

We investigated the effect of AS-IV on *β*-catenin expression in primary keratinocytes using two standard assays, namely immunofluorescence staining and Western blot analysis. As expected, keratinocytes incubated with EGF showed normal *β*-catenin membrane expression, but those treated with LiCl exhibited prominent nuclear *β*-catenin staining. LiCl-induced nuclear *β*-catenin was attenuated by AS-IV exposure, as evidenced by remarkably less *β*-catenin nuclear staining (Figure 6(a)). Similarly, Western blot analysis indicated that LiCl-treated keratinocytes expressed over 5-fold more *β*-catenin than the control cells. In addition, AS-IV exposure of LiCl-treated cells led to nearly 3-fold less *β*-catenin that that in the control group (Figure 6(b)) and additionally, PCNA, which indicating the cell proliferation, was downregulated 50% by LiCl compared with DMSO control, and upregulated to 80% by AS-IV (Figure 6(c)).

### 3.5. AS-IV Attenuates LiCl-Induced Migration Inhibition of Keratinocytes

**Figure 7:**
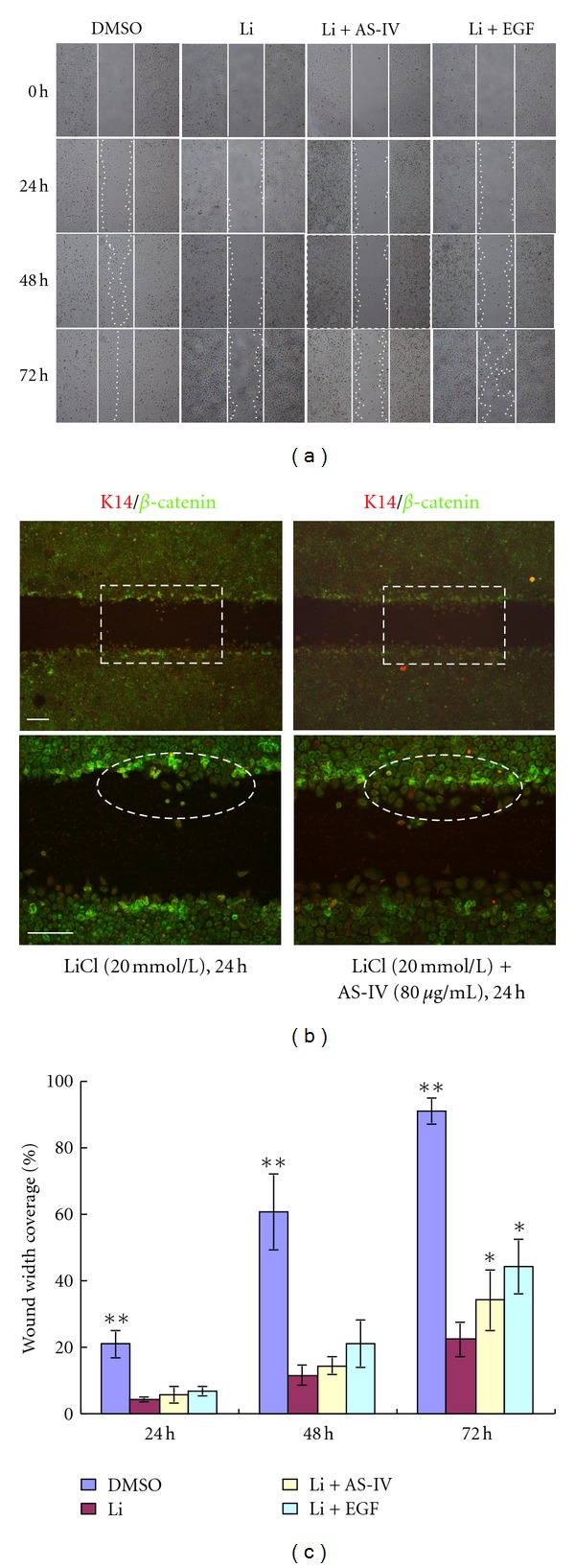
AS-IV attenuates LiCl-induced inhibition of keratinocyte migration. (a) Mouse primary keratinocytes with LiCl-induced activation of *β*-catenin were used in the scratch assay. LiCl inhibited keratinocyte migration compared to untreated cells. Inhibition was prominent at 48 h and was sustained through 72 h (**P < 0.01). EGF (positive control) stimulated migration was significant after 72 h, as evidenced by the nearly closed wound. AS-IV attenuated the LiCl-induced keratinocyte migration inhibition effect, as evidenced by the difference in wound closure after 72 h (*P < 0.05, compared with LiCl-treated group). Straight lines demarcate the initial wound area, and dotted lines indicate the migrating front of cells. (b) *β*-catenin was upregulated at the scratch wound edge. Insets show enlarged images of the dotted rectangle. The white circle indicates the keratinocytes starting to migrate from the nonnuclear *β*-catenin-positive cell. Scale bar = 100 *μ*m. (c) Histograms showing the average coverage of scratch wounds widths as percentages of the baseline wound width at time of scratch (0) and 24 h, 48 h, and 72 h after LiCl, LiCl + AS-IV, and LiCl + EGF treatments. *P < 0.05, **P < 0.01. Scale bar = 100 *μ*m.

To test if LiCl-induced overexpression of *β*-catenin can affect keratinocyte migration during wound healing, we used the *in vitro* wound scratch assay. Keratinocytes were incubated with LiCl, LiCl and EGF (positive control), and/or AS-IV for 72 h. Keratinocyte migration into the scratch area was observed and the distance was quantified. As shown in Figure 7, LiCl treatment inhibited keratinocyte migration by almost 80%, whereas LiCl and AS-IV promoted migration by 60%, presumably via downregulation of activated *β*-catenin.

### 3.6. AS-IV Attenuates LiCl-Induced Downregulation of *β*-Tubulin

**Figure 8:**
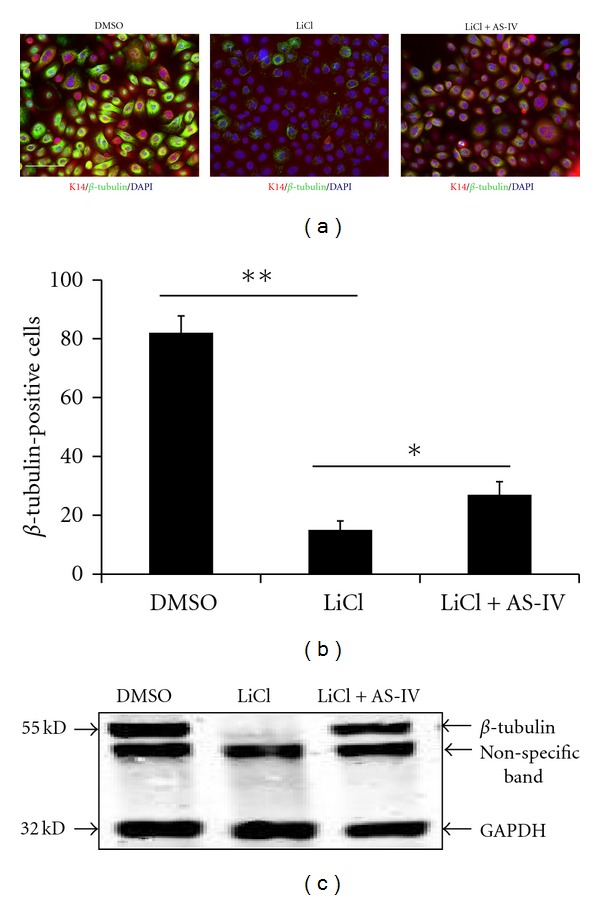
AS-IV attenuates LiCl-induced *β*-tubulin downregulation. Keratinocytes were treated with LiCl (20 mM) and LiCl (20 mM) + AS-IV (80 *μ*g/mL) for 24 h, and *β*-tubulin was determined by immunofluorescence (a, merged images of K14/*β*-tubulin/DAPI were shown) and immunoblotting ((c), GAPDH protein expression was used as the protein loading control). (b) Cell numbers of *β*-tubulin-positive keratinocytes per 100 total cells are presented. AS-IV significantly attenuated LiCl-induced *β*-tubulin downregulation. *P < 0.05, **P < 0.01. Scale bar = 100 *μ*m.

Tubulin protein is a key mediator of cell migration [16]. To determine whether LiCl treatment affected *β*-tubulin expression, we incubated primary keratinocytes with LiCl alone or with AS-IV. Changes in *β*-tubulin expression levels were evaluated by immunofluoresence following staining of treated cells with a *β*-tubulin-specific antibody (Figure 8(a)), and by immunoblotting (Figure 8(c)). *β*-tubulin was expressed in most of the untreated primary keratinocytes, but LiCl-treatment reduced *β*-tubulin expression. As shown in Figure 8(b), *β*-tubulin was expressed in only 15% of LiCl-treated cells but was expressed in 27% of cells treated with LiCl and AS-IV. In the DMSO-treated control cultures, 80% of cells expressed *β*-tubulin. Together, our findings indicate that LiCl is able to cause downregulation of *β*-tubulin, which was resolved, at least in part, by treatment with AS-IV. Thus, overexpression of *β*-catenin during the wound healing process may impair healing by reducing *β*-tubulin expression, and subsequently inhibiting keratinocyte migration. Since AS-IV can attenuate LiCl-induced downregulation of *β*-tubulin, it may represent a promising therapeutic agent for improving migration and promoting wound healing.

## 4. Discussion

In developed countries, 0.2 to 1% of the population is affected by chronic venous ulcers [17]. In China, it has been reported that approximately 1.5%–3.0% of surgical inpatients are hospitalized for chronic refractory wounds. Nonhealing chronic wounds not only decrease the quality of life of the sufferer and increase risk of further infection and death, but also represent a significant economic burden on the healthcare system. The experiments described in the present study show that AS-IV is able to repair chronic cutanous wounds through regulation of cell migration and cell proliferation.

 Among the many biological molecules known to influence wound healing, Wnt signaling is believed to be crucial, as it mediates skin development throughout life and can exert both positive and negative effects on the wound healing process. LiCl is a well-characterized inhibitor of GSK3, and as such can effectively activate Wnt signaling *in vitro* [10]. Numerous studies have shown that LiCl can elicit remarkably various effects on different cell types. For instance, LiCl was shown to stimulate proliferation of cultured erythrocytes via activation of the Wnt/*β*-catenin signaling pathway [18]. In addition, LiCl-stimulated proliferation of MCF-7 human breast cancer cells and caused a remarkable change in the expression of phosphoinositide metabolites [19]. In contrast, administration of LiCl suppressed prostate cancer cell proliferation by disrupting the E2F-DNA interaction and subsequent E2F-mediated gene expression [20]. LiCl also inhibited proliferation of the human esophageal cancer cell line, Eca-109, by inducing G2/M cell cycle arrest [21]. Mao et al. reported that LiCl treatment induced cell cycle arrest in G_2_/M in primary bovine aortic endothelial cells without affecting cell viability [22]. These studies demonstrate that LiCl can mediate a wide variety of cellular functions depending on the context. Here, we demonstrate that LiCl can induce cell proliferation and migration inhibition and cause S phase arrest in mouse primary keratinocytes (Figure 5).

 The important role of Wnt signaling pathway activation in wound healing is well recognized [23, 24]. The Wnt signaling pathway is known to regulate a variety of cell functions, including cell fate, polarity, and differentiation, via the canonical or *β*-catenin stabilization pathway and/or the planar cell polarity or noncanonical pathway [25]. In addition, Wnt signaling was previously shown to mediate proliferation and migration of fibroblasts and keratinocytes [26]. Keratinocytes, the major cellular component of the epidermis, have several critical roles in the wound healing process. The keratinocytes that appear at the nonhealing edges of chronic wounds have been shown to overexpress *β*-catenin protein and are believed to differ from normal, healthy keratinocytes.

 During the normal wound healing process, the re-epithelialization stage involves covering the wound surface with a layer of epithelium, which involves differentiation, proliferation, and migration of epidermal keratinocytes. Research has shown that overexpression of *β*-catenin and c-myc contributes to impaired wound healing by inhibiting keratinocyte migration and altering their differentiation.

 A previous study from our laboratory also indicated that a Chinese formula that included Radix astragali ingredients might be effective in regulating the Wnt signaling pathway [11]. However, the underlying mechanisms have remained ambiguous. AS-IV is the major phytochemical compound of the well-known Chinese herbal medicine Huangqi, which is extracted from the dried root of Radix astragali, and is a widely used herbal remedy in traditional Chinese medicine for the treatment of diabetes and inflammation [7]. Therefore, the goal of the present study was to determine the effect of AS-IV on keratinocyte proliferation and migration in wound healing.

 In this report, we show that treatment of primary keratinocytes with LiCl, an inhibitor of GSK-3*β* and an activator of the Wnt signaling pathway, induced stabilization and nuclear translocation of *β*-catenin associated with cell proliferation and migration inhibition. Moreover, LiCl treatment significantly induced cell cycle arrest in the S phase. MTS/PMS and cell viability assays showed that LiCl induced cell growth retardation without affecting cell viability; it appeared that this effect was a result of induction of a senescent-like phenotype. AS-IV treatment of LiCl-induced keratinocytes resulted in attenuated growth retardation and reduced amounts of cells in the S phase. These new findings led us to hypothesize that *β*-catenin overexpression during impaired wound healing may account for the dysfunctional keratinocyte proliferation and migration.

 In our study, the BrdU incorporation assay was used to determine DNA synthesis activity as an indirect method of evaluating cell proliferation. BrdU analysis showed that AS-IV treatment was able to increase the percentage of BrdU-positive cells (Figure 3(e)), indicating that AS-IV can effectively promote DNA synthesis activity in keratinocytes. Keratinocyte migration is essential for skin wound healing. Treatment with LiCl alone inhibited keratinocyte migration abilities in a wound scratch assay. Interestingly, *β*-catenin was upregulated at the scratch wound edge in LiCl-induced keratinocytes cultures. AS-IV effectively attenuated this LiCl-induced migration inhibition effect (Figure 7). Moreover, we found that keratinocytes started to migrate from the nonnuclear *β*-catenin positive cell, indicating that the migration of *β*-catenin-positive cells is blocked and AS-IV treatment improves cell migration associated with *β*-catenin expression.

 In summary, using LiCl as a tool to activate Wnt/*β*-catenin signaling, we have shown the functional relevance of Wnt/*β*-catenin signaling in keratinocyte proliferation and migration. Importantly, we have also provided evidence for the application of AS-IV in the treatment of wound healing and revealed its role in the wound healing reepithelialization stage. These AS-IV related discoveries provide novel insights into the molecular mechanisms underlying the significant regulation of the proliferative effects of AS-IV in keratinocytes, which can enhance epithelialization and healing of chronic wounds [27]. Future studies are needed to further elucidate the effect of AS-IV on other molecules and cells involved in wound healing, as this will help to advance the development of this drug for clinical application.

## Abbreviations

AS-IV: Astragaloside IV
LiCl: Lithium chloride
DMSO: Dimethyl sulfoxide
GSK-3: Glycogen synthase kinase-3
MTS/PMS: 3-(4,5-dimethylthiazol-2-yl)-5-(3-carboxymethoxyphenyl)-2-(4-sulfophenyl)-2H-tetrazolium, inner salt/phenazine ethosulfate
PI: Propidium iodide
BrDU: 5-bromo-2′-deoxyuridine
PBS: Phosphate buffered saline
IC_50_: Half maximal (50%) inhibitory concentration
PCNA: Proliferating cell nuclear antigen.
